# I speak fast when I move fast: the speed of illusory self-motion (vection) modulates the speed of utterances

**DOI:** 10.3389/fpsyg.2013.00494

**Published:** 2013-08-09

**Authors:** Takeharu Seno, Keiko Ihaya, Yuki Yamada

**Affiliations:** ^1^Faculty of Design, Kyushu UniversityFukuoka, Japan; ^2^Research Center for Applied Perceptual Science, Kyushu UniversityFukuoka, Japan; ^3^Institute for Advanced Study, Kyushu UniversityFukuoka, Japan; ^4^Faculty of Medical Sciences, Kyushu UniversityFukuoka, Japan; ^5^Research Institute for Time Studies, Yamaguchi UniversityYamaguchi, Japan

**Keywords:** vection, utterance

## Abstract

Speed of utterance is an important factor in smooth and efficient conversation. We report a technique to increase utterance speed and that might improve a speaker's impression and information efficiency in conversation. We used a visual display consisting of optic flows in a large visual field that induced participants' illusory self-motion perception (vection). The speed of vection corresponded to the speed of the optic flows. Using this method, we investigated whether vection speed affects utterance speed. We presented fast- and slow-moving optic flow stimuli while dynamically swapping random dots presented to participants, during which time the participants were asked to talk for 2 min. Results revealed that the utterance speed was significantly faster in the fast optic flow condition. Our method could be a stepping stone for establishing a technique of modulating speech speed effectively.

## Introduction

Utterance speed is an important factor in smooth and efficient conversation. In addition, it is known that utterance speed offers clues in estimating the personality of another person. For example, rapid utterances tend to increase the impression that the speaker has a high degree of competence (Smith et al., 1975) and as such tend to promote persuasion (Miller et al., 1976). Moreover, relatively slower utterance speeds will reduce the amount of information conveyed during conversation, even though in certain contexts they can suggest that the speaker has a calm and gentle personality. For people who live a modern, fast-paced lifestyle, such as businesspersons, it is often necessary to verbally send large amounts of information to listeners within a given period of time (e.g., in the situation of a telephone call). Hence, it clearly seems that the development of techniques to increase utterance speed would be beneficial to improve the impression left by the speaker on the listener, as well as the information efficiency in such conversations.

A number of methods for modulating utterance speed have been developed, such as the pacing board (Helm, 1979) and DAF [Delayed Auditory Feedback (Lee, 1950; Yates, 1963)]. The pacing board consists of a narrow board with seven one-foot long divisions. The speaker uses the board by pointing to a different division for each syllable being uttered. In DAF, delayed feedback of a speaker's own utterances is given to the speaker. These methods serve the purpose of reducing the overall speed of utterances. However, there are still no practically valid methods for increasing the speed of utterances. Moreover, the pacing board method requires speakers to always have their hands occupied, which can be inconvenient for making hand gestures. Therefore, hands-free methods for increasing utterance speed are needed. In this study, we attempted to develop a new technique based on visual presentation to increase the utterance speed of speakers. While our method also might limit speakers' behavior somewhat in terms of visual distraction, considering the fact that similar methods have already been used in information presentation technologies using augmented reality, e.g., projection onto the front glass of a car, our method should similarly be implementable with such technologies and optimized to minimize the visual load on speakers.

In the current study we focused on vection in which a class of motion perception. When stationary participants are exposed to a large visual motion field that simulates the retinal flow generated by self-translation or self-rotation, they often experience an illusory perception of self-motion; this phenomenon is known as vection (Fischer and Kornmüller, 1930). Vection is inherently susceptible to sensory processing in modalities other than vision. For example, vection has been facilitated by locomotion (Seno et al., 2011a) and by wind to the face (Seno et al., 2011b). Furthermore, consistent vestibular input (Wright, 2009), consistent head movements (Ash et al., 2011), and consistent somatosensory cues added to a hand also facilitate vection (Lécuyer et al., 2004). In addition, vection can be further facilitated by vibrations (subsonics) consistent with visual rotation (Riecke et al., 2008).

Note that vection and action are related, in particular with respect to speed. Although previous studies have not directly examined this relationship, considering the accumulated knowledge on the interplay between vection and other sensory processing, vection is likely to also interact with action. In a previous study, the speeds of visual stimuli and human action were reported; Watanabe (2007) reported that when participants watched fast-moving biological motion, their simple response time became shorter than when they observed slower biological motion, suggesting that, under certain circumstances, the speed of dynamic stimuli increases a participant's action speed. We hypothesized that this effect found by Watanabe could be expanded to other visual stimuli that induce not only the perception of object motion, but also self-motion. That is, the speed of visual stimuli comprising the optic flow may also affect action speed. We assume that an utterance represents a class of such actions; as such, it is possible that vection, which is induced by optic flows, affects utterance speed.

The present study aimed at investigating whether vection speed can modulate utterance speed. To this end, we employed fast and slow optic flow stimuli to induce fast and slow vection, respectively. In addition, dynamically swapping random dots that did not induce vection were used as control stimuli. We assumed that if the speed of vection governed the speed of utterance, then a participant's utterance speed would be accelerated when viewing the optic flow stimuli compared to the control stimuli. We hypothesized that the fast and slow optic flow conditions would accelerate utterance speed to a greater degree than the dynamic random dot condition, and that the degree of the modulation would be larger in the fast optic flow condition than in the slow condition.

## Methods

### Participants

Fifteen adult volunteers participated in the experiment. The participants were either graduate or undergraduate students, with no reported visual or vestibular abnormalities. All participants were naive as to the purpose of the present study.

### Apparatus and stimuli

Stimulus images were generated and controlled by a computer (Apple, MB543J/A). These stimuli were presented on a plasma display (3D VIERA, 50 inches; Panasonic) with a 1024 × 768 pixel resolution at a 60-Hz refresh rate. The experiments were conducted in a dark chamber. The viewing distance was 57 cm. An IC recorder (Roland, R-09HR) was used to record the speech of the participants.

In the experiment, we presented three types of visual stimuli: fast and slow optic flow stimuli, and dynamic random dots (DRD). These three stimulus types corresponded to fast vection, slow vection, and the absence of vection, respectively. We used optic flow stimuli involving expansion and contraction. Stimuli were created by randomly positioning 16,000 dots inside a simulated cube, and then moving the participant's viewpoint to simulate forward self-motion of 32 or 1 m/s, corresponding to the fast or slow optic flow conditions, respectively. In addition, DRD were presented at 0.1 Hz (1240 dots/frame). The velocities of the dots ranged from 0 to 45°/s in the fast vection condition and from 0 to 1.4°/s in the slow vection condition, and no velocity (0°/s) in the DRD condition. The results confirmed that both the fast and slow optic flow stimuli induced substantial vection, and that the DRD stimuli did not induce any vection. The participants were instructed to gaze at the center of the screen. While the gaze direction was not specifically recorded, no participants reported that their gaze highly deviated from the center of the screen.

### Procedure

In each trial, participants viewed each of the stimuli for the duration of the trial. All participants participated in all three experimental conditions. The order of conducting the three conditions was fully randomized. In each condition, the trial was repeated once. During stimulus presentation, participants were instructed to speak for 2 min on one of six topics provided by the experimenter. The topics were related to the self (hobbies, childhood, grade school days, university days, people they respected, and their personalities). Three of the six topics were randomly presented to the participants; specifically, the topic “childhood” was assigned to seven participants, “hobbies” to nine, “grade school days” to eight, “personality” to six, “respected person” to seven and “university days” was assigned to eight participants. The assigning of the six topics and the ordering of the three optic flow conditions were also randomized by the computer. All three conditions were successively conducted on the same day without a large temporal gap. The experimenter recorded all speech by an IC recorder. The experimenter initiated the speech by an oral cue such as “Please start.” The speech duration was defined as the 2-min period beginning from the point when the participants began to speak.

## Results

A third person who did not know the purpose of the experiment calculated the total duration of the speech, the total number of morae it contained, and the utterance speed (morae/sec). Mora is a unit in phonology that determines syllable weight, which in Japanese languages determines stress or timing. Moreover, a whole utterance disruption, which is the period without sound or meaningful voices, was also calculated. For example, sounds like “Ah” or “Uh” were included in the disruption. The speech was analyzed using Audacity (The Audacity Team) and Wavez (Osamu Kurai) software. The coding criteria for the audio data remained constant for the duration of all analyses.

**Figure 1:**
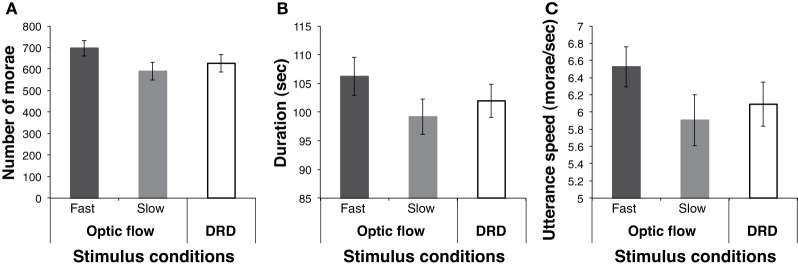
**Results of the experiment**. The results for **(A)** number of morae, **(B)** speech duration, and **(C)** utterance speed in each of the stimulus conditions are shown. The labels “Fast” and “Slow” represent the results of the fast optic flow and slow optic flow conditions, respectively. Error bars denote the standard errors of the mean.

As shown in Figure 1, in the fast optic flow condition, the speech duration, number of morae, and utterance speed showed the longest, largest, and fastest results, respectively, among the stimulus conditions. A One-Way analysis of variance (ANOVA) with stimulus condition as a within-subject factor revealed a significant main effect of the three conditions in the three measures [duration: *F*_(2, 14)_ = 4.14, *p* < 0.03, *p*_rep_ = 0.94, η^2^_*p*_ = 0.28; mora: *F*_(2, 14)_ = 9.26, *p* < 0.0009, *p*_rep_ = 0.99, η^2^_*p*_ = 0.40; speed: *F*_(2, 14)_ = 9.67, *p* < 0.0007, *p*_rep_ = 0.99, η^2^_*p*_ = 0.41]. Multiple comparisons using Ryan's method revealed that utterance speed was significantly higher in the fast optic flow condition than in the slow optic flow and DRD conditions (*p*s < 0.006). Moreover, there were significantly more morae in the fast optic flow condition than in the slow optic flow and DRD conditions (*p*s < 0.01). Fast vection induced fast utterance speed. Moreover, differences in duration between the fast and slow conditions, between the fast and DRD conditions, and between the slow and DRD conditions were significant (*p*s < 0.05).

Furthermore, we also calculated the average duration per mora. Then the results again showed that in the fast condition, duration of each mora was shortest in the fast optic flow condition. The mean values of duration/mora were 0.156 (*SD* = 0.022), 0.175 (*SD* = 0.032), and 0.268 (*SD* = 0.024) seconds for the fast, slow, and DRD conditions, respectively. A One-Way ANOVA revealed a significant main effect of the three conditions [*F*_(2, 28)_ = 8.42, *p* < 0.002, *p*_rep_ = 0.99, η^2^_*p*_ = 0.38]. *Post-hoc* multiple comparisons (Ryan's method) revealed significant differences between the fast and the other two conditions [fast vs. slow: *t*_(28)_ = 4.07, *p* < 0.0004, Cohen's *d* = 2.18; fast vs. DRD: *t*_(28)_ = 2.51, *p* < 0.02, Cohen's *d* = 1.34] but there was no significant difference between the slow and DRD conditions [*t*_(28)_ = 1.55, *p* > 0.13, Cohen's *d* = 0.83]. These results clearly indicated that speakers produced each mora more quickly in the fast condition.

We speculate that the reason not only the number of morae but also the duration increased in the fast vection condition is because the fast vection condition might have activated language-processing mechanisms in the brain, which then induced a faster utterance speed, resulting in the differences observed in each utterance index.

We calculated the number of morae and duration corresponding to each topic. Results showed that the values were approximately 660 and 100, respectively, for all six topics. For example, for the topic “childhood,” the values for the mean number of morae and duration were 664.8 (*SD* = 181.7) and 103.2 (*SD* = 10.4), respectively. We then conducted One-Way ANOVAs for these two factors, number of morae and duration, which revealed that there was no significant main effect of the six topics both with respect to the number of morae and duration [mora: *F*_(5, 39)_ = 0.15, *p* > 0.97, *p*_rep_ = 0.51, η^2^_*p*_ = 0.02; duration: *F*_(5, 39)_ = 0.18, *p* > 0.96, *p*_rep_ = 0.51, η^2^_*p*_ = 0.02]. Furthermore, there was no significant difference in utterance speed across the six topics [*F*_(5, 39)_ = 0.33, *p* > 0.89, *p*_rep_ = 0.54, η^2^_*p*_ = 0.04]. Taken together, these results show that there was neither a positive nor negative effect for any aspect of the utterances with respect to the six different topics.

There was also the possibility that faster utterances occurred at the expense of fluency and intelligibility. We thus conducted an additional experiment in which the fluency and intelligibility of each speech sample were evaluated by naive volunteers other than the participants in the abovementioned main speech experiment. Nine additional participants listened to the 2-min speech recordings made by the participants in the main experiment, and then they evaluated the fluency and intelligibility of the utterances using an 11-step scales (from 0, not fluent/intelligible at all, to 10, very fluent/very intelligible). Results showed that subjective intelligibility did not differ across the three experimental conditions. The obtained values of subjective intelligibility for the three conditions were as follows: fast (*M* = 6.31, *SD* = 1.27), slow (*M* = 6.07, *SD* = 1.51), and DRD (*M* = 6.21, *SD* = 1.12). A One-Way ANOVA revealed no significant main effect for the three conditions [*F*_(2, 16)_ = 1.97, *p* > 0.17, *p*_rep_ = 0.83, η^2^_*p*_ = 0.20]. Conversely, subjective fluency differed across the three conditions; it was the highest (*M* = 6.09, *SD* = 1.56) in the fast vection condition, not as high (*M* = 5.90, *SD* = 1.63) in the DRD condition, and the lowest (*M* = 5.74, *SD* = 1.63) in the slow vection condition. A One-Way ANOVA revealed a significant main effect for the three conditions [*F*_(2, 16)_ = 14.48, *p* < 0.0003, *p*_rep_ = 0.99, η^2^_*p*_ = 0.64]. Multiple comparisons revealed significant differences between the fast and slow [*t*_(16)_ = 5.38, *p* < 0.0001, Cohen's *d* = 3.80], the fast and DRD [*t*_(16)_ = 2.92, *p* < 0.02, Cohen's *d* = 2.05], and the slow and DRD [*t*_(16)_ = 2.48, *p* < 0.03, Cohen's *d* = 1.75] conditions. In both of the evaluations, the fast vection condition did not result in the smallest obtained values, indicating that the observed fast speech was not produced at the expense of fluency and intelligibility. Thus, through our rating method, we successfully showed that fast speech was not produced at the expense of fluency and intelligibility.

Furthermore, to exclude the possibility of degraded fluency and intelligibility using a more objective method of analysis, we calculated the total duration of disruptions in each speech sample. The mean values of disruptions were 20.87 (*SD* = 10.86), 25.64 (*SD* = 10.65), and 23.30 (*SD* = 11.18) seconds for the fast, slow, and DRD conditions, respectively. A One-Way ANOVA revealed a marginally significant main effect of the three conditions in the total disruption duration [*F*_(2, 28)_ = 3.33, *p* = 0.0504, *p*_rep_ = 0.92, η^2^_*p*_ = 0.19]. Although main effect did not reach significance, the *p*-value was quite close to the significance level (α = 0.05), and hence we conducted *post-hoc* multiple comparisons to reveal further the differences between the conditions. The multiple comparisons (Ryan's method) revealed that there was a significant difference between the fast and slow conditions [*t*_(28)_ = 2.58, *p* < 0.02, Cohen's *d* = 1.38] but that there were not significant differences between the fast and DRD [*t*_(28)_ = 1.31, *p* > 0.19, Cohen's *d* = 0.70] and between the DRD and slow conditions [*t*_(28)_ = 1.27, *p* > 0.21, Cohen's *d* = 0.68] indicating that speakers less paused in the fast condition, although the effect size was relatively small. These results did not contradict our main finding that the utterance speed increased. We speculated that both utterance and disruption are mediated by a unitary mechanism and that the mechanism might be modulated by the fast vection and then both utterance and disruption simultaneously changed.

## Discussion

In this study, we attempted to develop a method to modulate utterance speed by visual stimulation that induced vection. To this end, we tested whether vection speed affected utterance speed. Two visual displays—with different optic flow speeds—were used to modulate utterance speed; one display induced fast vection, and the other induced slow vection. We predicted that both of these vection displays would induce faster utterances than a non-vection DRD display, and that the fast vection display would induce faster utterance speed than the slow display. Results partially proved our prediction: while the faster vection significantly accelerated utterance speed, no such effect of the slow vection display was obtained. This result might be related to the fact that the slow vection stimuli were not much different from the randomly generated, non-vection DRD display.

One might argue that some of the topics used as speech prompts for the participants were easier to discuss in the fast optic flow condition than in the slow optic flow condition, thereby resulting in more fluent utterances (i.e., more morae and faster utterances in the fast optic flow condition). However, in our experiment the topics were randomly chosen for each condition for each participant. Thus, if there was a bias in the difficulty of speech related to the topic, then this manipulation counterbalanced any such bias. Therefore, the possibility that some topics induced faster utterances should be negligible in the present experiment. In addition, we proved that there was no positive or negative effect of six different topics in the utterance speed.

The nature of the mechanism underlying our findings poses an intriguing question. One possible explanation is related to cognitive or semantic modulation. Semantic and cognitive representations of “fast” may be consistent across utterance speed and self-motion. This semantic connection might have been activated in the participants' minds during the experiment, yielding the current results. We previously reported that upward vection induced positive memories (Seno et al., 2013) and also that positive sounds enhanced upward vection (Sasaki et al., 2012). Thus, there is evidence that semantic-cognitive consistency, i.e., the connection of semantic representations of “upward” and “positivity,” can modulate both vection and cognition. A similar type of modulation also likely occurred in the present study, i.e., in the modulation of utterance speed. However, this account cannot explain why the present study found the effect of vection only in cases of acceleration.

It is also possible that the acceleration effect we observed is related to arousal level, especially if fast vection stimuli increased the participants' level of arousal. In previous studies of time perception, the notion of an “internal clock” has been proposed as a general pacemaker that governs the temporal aspects of human perception and action (Gibbon et al., 1984; Meck, 2005). The neural basis of this internal clock-like time measurement system has been debated (Gibbon et al., 1997; Mauk and Buonomano, 2004; Buhusi and Meck, 2005; Meck, 2005; Lewis and Miall, 2006). Furthermore, a number of previous studies have reported the effects of arousal on the internal clock (Droit-Volet and Meck, 2007), with increased arousal levels speeding up the internal clock. Our results suggest that this arousal-based speeding up of the internal clock may have modulated mental tempo, causing an increase in the participants' utterance speed. The increased speed of the internal clock was not consciously perceived, and as such it subconsciously altered (accelerated) the tempo of actions (utterances); thus, it is unlikely that participants consciously slowed down their utterance speed based on a conscious awareness of their increased utterance speed. In another study, we also revealed that vection could modulate arousal levels and mental tempo (Ihaya et al., submitted). Therefore, this account may explain why only the acceleration effect was observed, that is, why the arousal levels evoked by the optic flow were comparable in the slow and DRD stimuli. This account is more plausible than the cognitive account discussed above. Moreover, our results also suggest a number of other possibilities. For example, there is the possibility that the fast vection condition caused an increase in mental stress, thus inducing faster utterances. Future work should examine these possibilities in more detail and propose additional valid explanations.

The acceleration effect observed in the present study raises a number of interesting questions that should be topics of future research. For example, how long do these effects last? Are there marked individual differences? What are the maximal stimulus speed and exposure durations required to generate maximum effects? Understanding these points should lead to the development of not only instant adjustment techniques, but also techniques for the learning or preadjustment of utterance speed; that is, if a speaker is worried about his or her utterance speed, then such techniques would serve to increase the speed of all the processes involved before the actual speaking situations due to the long-lasting acceleration effect.

Other avenues for future research include the examination of whether other types of illusory self-motion (such as auditory vection and vestibularly-simulated self-motion) can also modulate utterance speed. If this turns out to be the case, then our proposed method will be applicable to even the visually impaired. Thus, as the first step to develop a technique for improving utterance speed, the present study would also be a steppingstone to further establish effective and easy-to-use techniques required for the practical treatment of a variety of clinical problems related to slow uttering speeds.

### Conflict of interest statement

The first and the third authors are aided by Japan Society for Promotion of Science. The first author is supported by Funds for the Development of Human Resources in Science and Technology (Japan Science and Technology Agency). This work is supported by Program to Disseminate Tenure Tracking System, Ministry of Education, Culture, Sports, Science and Technology, Japan. The other author declares that the research was conducted in the absence of any commercial or financial relationships that could be construed as a potential conflict of interest.
